# Quantitative Study of Non-Linear Convection Diffusion Equations for a Rotating-Disc Electrode

**DOI:** 10.3390/e25010134

**Published:** 2023-01-09

**Authors:** Fahad Sameer Alshammari, Hamad Jan, Muhammad Sulaiman, Din Prathumwan, Ghaylen Laouini

**Affiliations:** 1 Department of Mathematics, College of Science and Humanities in Alkharj, Prince Sattam bin Abdulaziz University, Al-Kharj 11942, Saudi Arabia; 2 Department of Mathematics, Abdul Wali Khan University, Mardan 23200, Pakistan; 3 Department of Mathematics, Faculty of Science, Khon Kaen University, Khon Kaen 40002, Thailand; 4 College of Engineering and Technology, American University of the Middle East, Egaila 54200, Kuwait

**Keywords:** mathematical modeling, non-linear equations, numerical solutions, hydrogen and hydroxide ion concentration, neural networks, machine learning, rotating-disc electrode, entropy

## Abstract

Rotating-disc electrodes (RDEs) are favored technologies for analyzing electrochemical processes in electrically charged cells and other revolving machines, such as engines, compressors, gearboxes, and generators. The model is based on the concept of the nonlinear entropy convection-diffusion equations, which are constructed using semi-boundaries as an infinite notion. In this model, the surrogate solutions with different parameter values for the mathematical characterization of non-dimensional $OH^{-}$ and $H^{+}$ ion concentrations at a rotating-disc electrode (RDE) are investigated using an intelligent hybrid technique by utilizing neural networks (NN) and the Levenberg–Marquardt algorithm (LMA). Reference solutions were calculated using the RK-4 numerical method. Through the training, validation, and testing sampling of reference solutions, the NN-BLMA approximations were recorded. Error histograms, absolute error, curve fitting graphs, and regression graphs validated the NN-BLMA’s resilience and accuracy for the problem. Additionally, the comparison graphs between the reference solution and the NN-BLMA procedure established that our paradigm is reliable and accurate.

## 1. Introduction

Rotating-disc electrodes (RDEs) allow performing steady-state studies of a redox reaction and measuring its kinetic parameters. The mass transfer rate may be controlled and enhanced in electrochemical investigations using hydrodynamic techniques or microelectrodes. Mass transport conditions can be easily changed to resolve (electro) chemical phenomena of various kinetics, such as electron transfers, adsorption/desorption processes, and coupled chemical reactions. Over the years, many hydrodynamic techniques (rotating disc/ring, channel, wall-jet, and dropping mercury electrodes) have been developed and used to explore the most common reaction mechanisms, such as EC, EC’, and ECE/DISP. The most widely used technique [1,2], for which a great deal of theoretical work has been done, is the rotating-disc electrode (RDE). For the first time, Levich solved the transient equation of diffusion for a spinning-disc electrode, which motivated the mathematical community to focus on the entropy and kinetics of electrode processing based on transport theories [3,4]. For proper geometries, the Navier–Stokes equation and the convection-diffusion equation solution are used to generate the mathematical models [3,4,5]. In fluid mechanics, the Von Kármán whirling viscous flow issue is well-known. Von Kármán’s original problem concerns a viscous flow caused by an infinitely revolving disc in a situation where the fluid far from the disc is at rest. Von Kármán first investigated steady laminar flows of a viscous Newtonian fluid through an infinite spinning disc [6,7]. The equations of Navier–Stokes are converted to ordinary differential equations using an ingenious similarity transformation that was also introduced by Von Kármán [6,8]. The momentum integral approach was then used to solve the problems. The numerous electrocatalytic processes have all been extensively studied using rotating-disc electrodes. Unraveling reactions with rotating electrodes by Bruckenstein describe how the RDE and allied techniques can be used to unravel complicated heterogenous and homogenous reactions [9]. Popovic described a ring-disk study of the competition between anodic oxygen transfer and dioxygen-evolution reactions [10]. Electrocatalysis of anodic oxygen-transfer reactions: chronoamperometric and voltammetric studies of the nucleation and electrodeposition of β-lead dioxide at a rotating gold disk electrode in acidic media were performed by Change [11]. Treimer presents the comparison of voltammetric responses of toluene and xylenes at iron (III)-doped, bismuth (V)-doped, and undoped β-lead-dioxide film electrodes and consideration of the application of Koutecky–Levich plots in the diagnoses of charge-transfer mechanisms with rotated disk electrodes [12,13]. Electro-oxidation of aqueous p-methoxy phenol on lead oxide electrodes was presented by Borras [14]. A novel mounting methodology for cylindrical samples for use as spinning-disc electrodes was created by Cahan et al. [15,16], which solves numerous issues with more traditional methods. Below the limiting current, Newman [17,18] attained the uniform current density on a revolving disc electrode. Eddowes et al. [19,20] used the orthogonal collocation and finite-difference techniques to resolve the spinning-disc electrode system. Both methods reduce the ordinary differential equation into a group of concurrent equations that can be solved with a single matrix operation. Nolan et al. [21,22] examined the first-order EC-catalytic process at RDE using polynomial approximation. At RDE, Nolan et al. [23,24] also found a 2nd-order EC-catalytic iterative solution. To measure the concentration on the revolving disc electrode in both transient and steady-state settings, the homotopy perturbation technique was employed by Jansi Rani et al. [25,26]. Using fluid viscosity, Chitra et al. [27,28] computed the steady-state output of spinning disc flow associated with the mass-concentration field. Dong et al. [29,30] carried out the numerical simulations of a two-dimensional axisymmetric cell with a revolving disc electrode. The generation of electrochemical hydrogen at RDE was modeled mathematically by Grozovski et al. [31,32]. Recently, an equation for the production of hydrogen at a revolving disc electrode (RDE) was created by Sylvia et al. [33,34].

- This study’s primary goals were to analyze a mathematical model for the reduction of $H^{+}$ ions and electrolysis of $H_{2}O$ in non-buffered aqueous electrolyte solutions and to investigate how specific parameters affect the e entropy of hydrogen ($H^{+}$) and hydroxide ($OH^{-}$) ions in a rotating-disc electrode (RDE).
- The mathematical model of the convection-diffusion equation for the non-dimensional hydrogen ($H^{+}$) and hydroxide ($OH^{-}$) ion concentrations on a rotating-disc electrode (RDE) has been solved for this problem.
- The behavior of the hydrogen ($H^{+}$) and hydroxide ($OH^{-}$) ion concentrations are studied using the backpropagated Levenberg–Marquardt algorithm (BLMA) and neural networks (NNs).
- The reference data of target solutions were produced by the Runge–Kutta technique and were successfully used in the supervised learning phase of the NNs-BLMA.
- Convergence analysis based on curve fitting, mean-square error, error histograms, and regression analysis by reference data was used to verify the effectiveness of the designed NN-BLMA. The results establish that the suggested method is slick and straightforward, extending to more complex problems.

## 2. Mathematical Formulation of the Problem

As long as the transfer is only produced by convection and diffusion, the transmission and entropy of numerous physical quantities, such as energy and particles, may often be explained using the convection-diffusion equation. The basic form of the convection-diffusion equation is

(1) $$\frac{\partial C}{\partial t}+v.\nabla c=D\nabla^{2}c,$$

(2) $$\frac{\partial C}{\partial t}=D\nabla^{2}c-v.\nabla c,$$

where *v* represents the velocity of the electrolyte, *c* stands for the concentration of diffusing species, *D* is the coefficient of diffusion, and $\nabla^{2}$ is the Laplacian operator. In one dimensional form, Equation (2) can be condensed into [35,36]

(3) $$\frac{\partial C_{i}}{\partial t}=D_{c_{i}}\frac{\partial^{2}C_{i}}{\partial z^{2}}-v_{z}\frac{\partial C_{i}}{\partial z},$$

where $C_{i}$ denotes species concentration, $v_{z}$ denotes the fluid velocity, and $D_{c_{i}}$ is the corresponding coefficient of diffusion. $H^{+}$ reduction in acidic solutions can result in the formation of hydrogen:

(4) $$H^{+}+e^{-}\to \frac{1}{2}H_{2},$$

The electroreduction of water itself is the main source of hydrogen in solutions with a pH > 7:

(5) $$H_{2}O+e^{-}\to \frac{1}{2}H_{2}+OH^{-},$$

In this study, the hydrogen evolution reaction using numerical simulations on a rotating-disc electrode (RDE) submerged in firmly supported, unbuffered fluids at various pH levels is described. Two distinguishing portions may be seen in the stationary polarization curves obtained in mildly acidic solutions; Equation (4) is related to the electro reduction of $H^{+}$, and Equation (5) is primarily concerned with the electrolysis of water. Due to the rapid recombination of $H^{+}$, a reactant of Equation (4), and $OH^{-}$, a product of Equation (5), considering these processes independently is not a solid technique for characterizing the entire mechanism:

(6) $$H^{+}+OH^{-}⇌H_{2}O.$$

**Figure 1:**
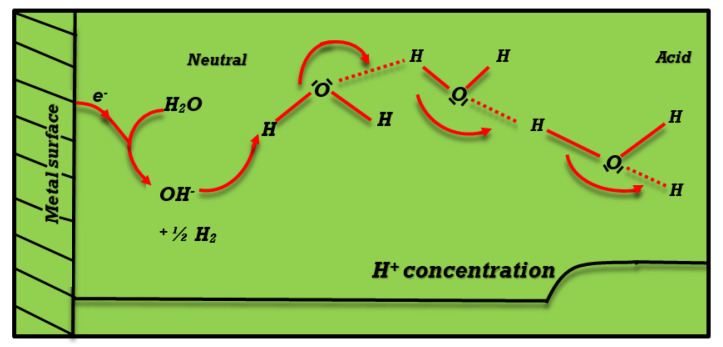
Diagram illustrating the electrolysis of $H_{2}O$ and the reduction of $H^{+}$ ions in nonbuffered aqueous electrolyte solutions.

In addition to determining steady-state pH profiles corresponding to certain electrode potentials, Equation (6) must be taken into account for the modest fluctuation of the “limiting” $H^{+}$ reduction current with the electrode potential [37,38], Figure 1 illustrates this system of reaction.

The $H^{+}$ and $OH^{-}$ concentrations inside the system may be represented by the mass balance equation as follows [33,39]:

(7) $$D_{H^{+}}\frac{d^{2}}{dz^{2}}C_{H^{+}}\left(z\right)+k_{-3}=v_{z}\frac{d}{dz}C_{H^{+}}\left(z\right)+k_{+3}C_{H^{+}}\left(z\right)C_{OH^{-}}\left(z\right),$$

(8) $$D_{OH^{-}}\frac{d^{2}}{dz^{2}}C_{OH^{-}}\left(z\right)+k_{-3}=v_{z}\frac{d}{dz}C_{OH^{-}}\left(z\right)+k_{+3}C_{H^{+}}\left(z\right)C_{OH^{-}}\left(z\right),$$

where $D_{H^{+}}$ is the coefficient of diffusion of $H^{+}$ ions and $D_{OH^{-}}$ is the coefficient of the diffusion of $OH^{-}$ ions. $C_{H^{+}}$ (z) is the concentration of $H^{+}$ ions, and $C_{OH^{-}}$ (z) is the concentration of $OH^{-}$ ions. $k_{-3}$ and $k_{+3}$ are the backward and forward reaction rate coefficients for Equation (6). At this stage, it was assumed that the transfer of mass occurs only by diffusion and convection, and other modes of transportation are disregarded. Regardless of concentrations and spatial coordinates, the diffusion coefficients $D_{H^{+}}$ and $D_{OH^{-}}$ are also assumed to be constants. The Cochran series solution of the Von Kármán equations may be used to characterize the composition of the fluid velocity $v_{z}$ [6,35,40]:

(9) $$v_{z}=-0.51023\;v^{\frac{-1}{2}}\Omega^{\frac{3}{2}}z^{2}+\frac{1}{3}\;v^{-1}\Omega^{2}z^{3}+\ldots ,$$

with Ω being the angular velocity of the electrode and v being the kinematic viscosity of the electrode. For the majority of solvents (Schmidt number (Sc) ≥ 100 [41,42]), an appropriate description is obtained by taking into account the first two terms in Equation (9). Injecting the first two components of the Cochran expansion into Equation (3) results in

(10) $$\frac{\partial C_{i}}{\partial t}+\left(-0.51023\;v^{\frac{-1}{2}}\Omega^{\frac{3}{2}}z^{2}+\frac{1}{3}\;v^{-1}\Omega^{2}z^{3}\right)\frac{\partial C_{i}}{\partial z}=D_{c_{i}}\frac{\partial^{2}C_{i}}{\partial z^{2}},$$

and the initial and boundary conditions are

(i) At z = 0, the two species become
  (11) $$at\;z=0,\;\frac{dC_{H^{+}}}{dz}=C_{H^{+}}^{\infty }\;and\;\frac{dC_{OH^{-}}}{dz}=0,$$
(ii) As z →∞, the concentration of $H^{+}$ ions ($C_{H^{+}}$) equals the bulk concentration of $H^{+}$ ions ($C_{H^{+}}^{\infty }$), and the concentration of $OH^{-}$ ions ($C_{OH^{-}}$) approaches zero. That is,
  (12) $$as\;z\to \infty ,\;C_{H^{+}}=C_{H^{+}}^{\infty }\;and\;C_{OH^{-}}\to 0,$$
(iii) The $H^{+}$ and $OH^{-}$ concentrations become
  (13) $$C_{H^{+}}\left(0,t\right)=e^{\eta }C_{OH^{-}}\left(0,t\right),$$
  (14) $$D_{H^{+}}{\left(\frac{dC_{H^{+}}}{dz}\right)}_{z=0}=-D_{OH^{-}}{\left(\frac{dC_{OH^{-}}}{dz}\right)}_{z=0},$$
  where η is potential, which is equal to
  (15) $$\eta =\frac{F}{RT}\left(E-E^{0^{{}^{\prime }}}\right),$$
  where $E^{0^{{}^{\prime }}}$ is the formal potential; *E* is the applied potential; and *F*, *R*, and *T* have their standard meanings [35,43].

When the previously described problem is resolved, and the concentration profiles are known, the current response (*i*(*t*)) is established as

(16) $$\frac{i(t)}{FA}=-D_{H^{+}}{\left(\frac{dC_{H^{+}}}{dz}\right)}_{z=0},$$

as long as the diffusion rates of the two electroactive species are equivalent ($D_{H^{+}}$ = $D_{OH^{-}}$= *D*). It is feasible to demonstrate that the sum of electroactive species’ concentrations stays constant throughout the experiment in any part of the solution, which implies: $C_{H^{+}}$(z,t) + $C_{OH^{-}}$(z,t) = $C_{H^{+}}^{\infty }$. The surface concentrations of the electroactive species are immediately found by combining this result with the Nernstian condition in Equations (13) and (14):

(17) $$C_{H^{+}}\left(0\right)=C_{H^{+}}^{\infty }\frac{e^{\eta }}{1+e^{\eta }}\;and\;C_{OH^{-}}\left(0\right)=C_{H^{+}}^{\infty }\frac{1}{1+e^{\eta }},$$

Moreover, it is noted that

(18) $$C_{H^{+}}C_{OH^{-}}=10^{-14}mol^{2}dm^{-6}.$$

Equations (7) and (8) can be rewritten in the non-dimensional form as follows:

(19) $$\frac{d^{2}m}{d\zeta^{2}}+\zeta^{2}\frac{dm}{d\zeta }-c_{1}mn+c_{0}=0,$$

(20) $$\frac{d^{2}n}{d\zeta^{2}}+\zeta^{2}\frac{dn}{d\zeta }-c_{1}mn+c_{0}=0,$$

where the non-dimensional parameters are 

m = $\frac{C_{H^{+}}}{C_{H^{+}}^{\infty }}$, n = $\frac{C_{OH^{-}}}{C_{H^{+}}^{\infty }}$, $c_{0}$ = $\frac{k_{-3}}{D^{\frac{1}{3}}a^{\frac{2}{3}}C_{H^{+}}^{\infty }}$, $c_{1}$ = $\frac{k_{+3}C_{H^{+}}^{\infty }}{D^{\frac{1}{3}}a^{\frac{2}{3}}}$, ζ = z ($\frac{a}{D}{)}^{\frac{1}{3}}$, and

(21) $$a=0.51023\;\varpi^{\frac{3}{2}}v^{\frac{-1}{2}}.$$

From Equation (18), the values of m and n are attainable as follows:

(22) $$mn=\left(10^{-14}mol^{2}dm^{-6}\right){\left(C_{H^{+}}^{\infty }\right)}^{2},$$

(23) $$let\;c=-c_{1}mn+c_{0},$$

Equations (19) and (20) become

(24) $$\frac{d^{2}m}{d\zeta^{2}}+\zeta^{2}\frac{dm}{d\zeta }+c=0,$$

(25) $$\frac{d^{2}n}{d\zeta^{2}}+\zeta^{2}\frac{dn}{d\zeta }+c=0;$$

the dimensionless initial and boundary conditions become

(26) $$At\;\zeta =0,\;\frac{dm}{d\zeta }=1\;and\;\frac{dn}{d\zeta }=0,$$

(27) Atζ=1,m=1andn=0,

(28) $$At\;\zeta =0,\;m=ne^{\psi }.$$

Equations (19) and (20) are a set of ordinary inhomogeneous differential equations that are severely nonlinear. To solve these equations numerically, the finite difference [19,44] and the orthogonal collocation [21,45] techniques can be applied.

The artificial neural network (ANN), a machine learning technique that focuses on the supervised neural processes, is discussed in this model created by McCulloch, based on the human brain in 1943. ANNs can learn, recognize, and deal with a wide range of complicated issues. Feed-forward neural networks (FFNNs) are the only ANN models that are widely used in a wide range of applications. A neural network (ANN) is a linked neuron network that can process several inputs but produces only one output. This work uses a multiple-layer perceptron (MLP) to optimize the number of hidden units. The MLP, sometimes referred to as the feed-forward neural network (FNN), is a form of neural network that contains a hidden layer between the input and output layers. The architectural depiction of an FFNN makes it interesting, since it enables the identification of a computational model (a function) in network form. Furthermore, an FFNN framework makes it a popular function approximator. It has the effect of approximating and solving any function or challenge. The connection weights and biases were also optimized. The standard MLP construction with one hidden layer is as follows:

(29) $$A_{j}=\sum_{i=1}^{n}w_{ij}x_{i}+b_{j},$$

where $x_{i}$ denotes inputs, $b_{j}$ denotes biased vectors, and $w_{ij}$ denotes connection weights, respectively. The activation function, a log-sigmoid, is used in the feed-forward neural network model, which is expressed as:

(30) $$f_{j}\left(x\right)=\frac{1}{1+e^{-Aj}}.$$

**Figure 2:**
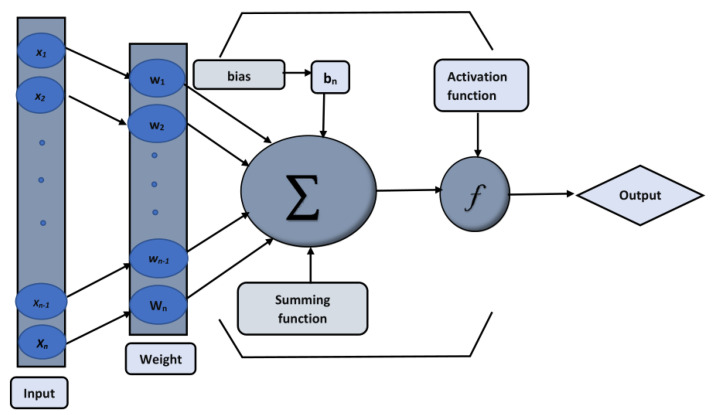
The architecture of a single neural network.

**Figure 3:**
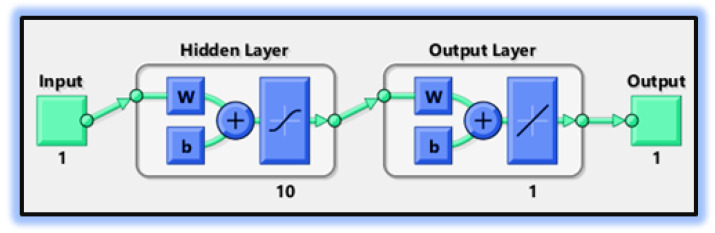
The architecture of a neural network.

- In the first step, a numerical solution is computed using the Runge–Kutta technique of fourth order ($Rk_{4}$) using Mathematica’s “ND Solve” module to create an initial dataset.
- In the second step, using the “nftool” from the MATLAB package, the BLM algorithm is run with the proper hidden neuron parameters and test data. Additionally, BLM employs the training, testing, and validation process and a reference solution to provide approximations for various nonlinear equation instances. Figure 2 and Figure 3 illustrate the NNs-LM technique using a single neuron model.

**Figure 4:**
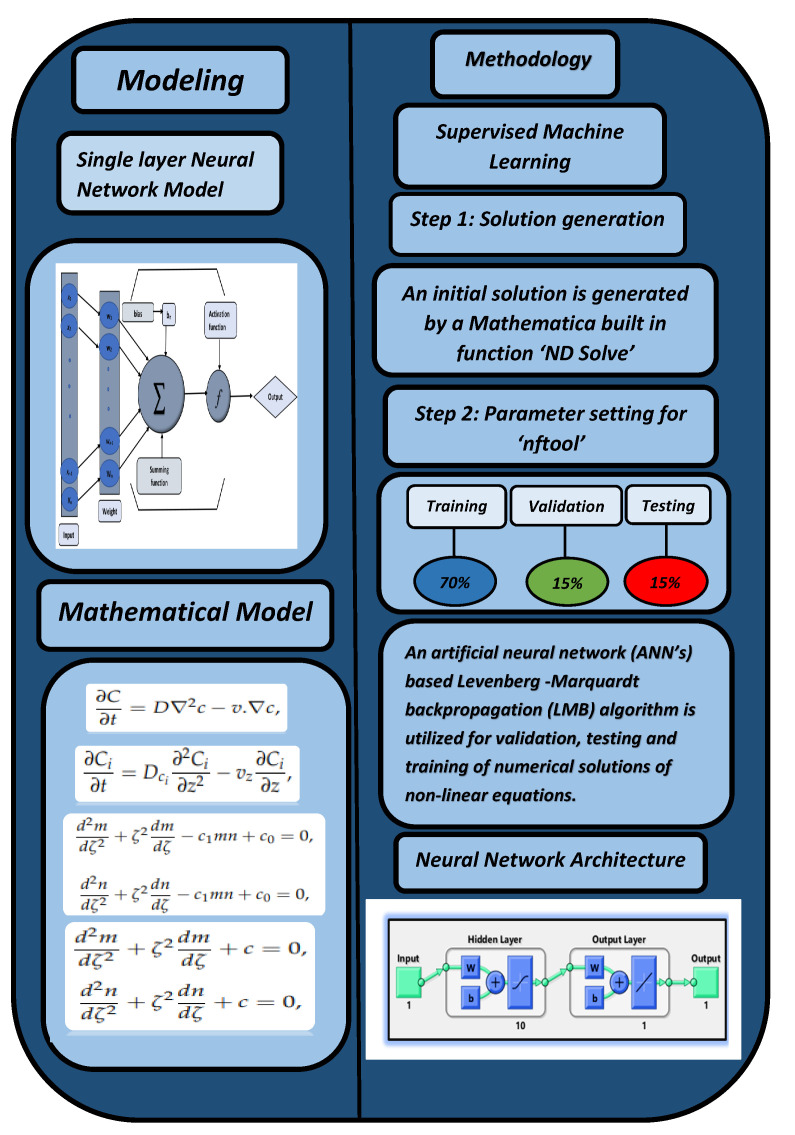
Mechanism of the NN-BLMA for the solution of highly nonlinear equations.

A two-step process is used to implement NN-BLMA. Figure 4 presents the design algorithm’s detailed workflow.

## 3. Comparison of Numerical Solutions

**Figure 5:**
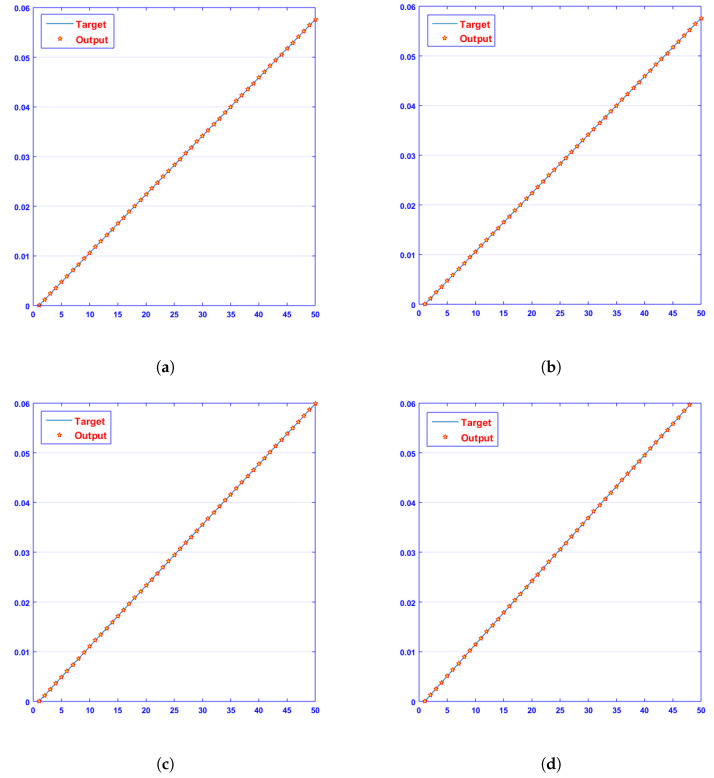
Results of dimensionless $H^{+}$ ion concentration with various rate constants, c. (**a**) at c = 0.1; (**b**) at c = 0.2; (**c**) at c = 0.3; (**d**) at c = 0.4.

**Figure 6:**
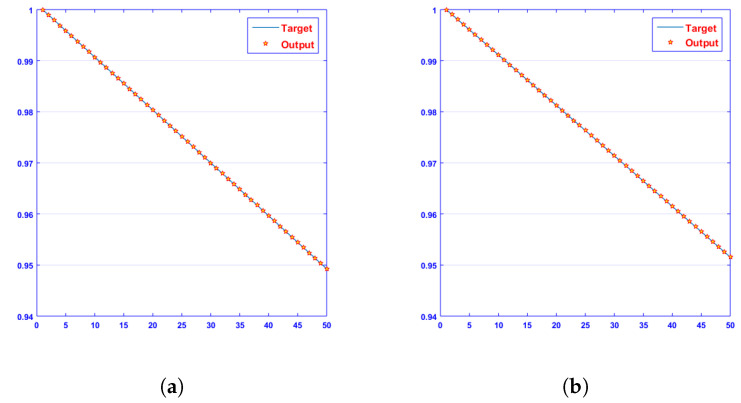

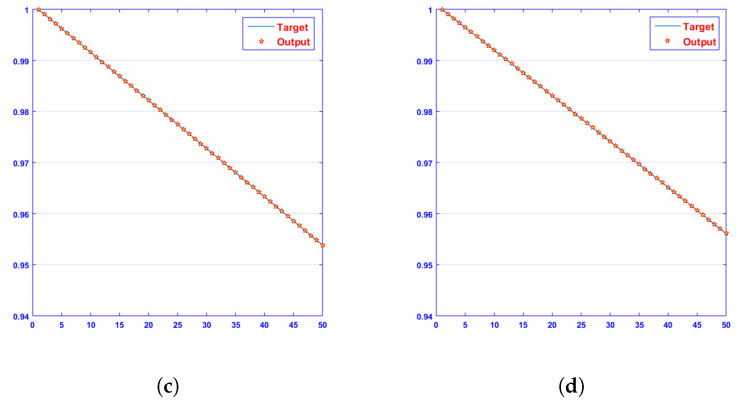
Results of dimensionless $OH^{-}$ ion concentration with various rate constants, c. (**a**) at c = 0.1; (**b**) at c = 0.2; (**c**) at c = 0.3; (**d**) at c = 0.4.

The approximate solutions obtained by a designed algorithm, NN-BLMA, were compared with $Rk_{4}$’s results, which show the analysis of the phase plane between dimensionless $H^{+}$ and $OH^{-}$ ion concentrations. It contains 50 points in each case on the *y* axis. The comparison graphs are closer to the real solution of a surrogate model. The blue line represents targeted data, and red stars with yellow in the center represent the output data of a present surrogate model. Figure 5 demonstrates that at c = 0.1 and c = 0.3, the absolute error ranges between $10^{-7}$ and $10^{-10}$; at c = 0.2 and c = 0.4, it ranges between $10^{-7}$ and $10^{-8}$. Figure 6 demonstrates that at c = 0.1 and c = 0.3, the absolute error ranges between $10^{-7}$ and $10^{-10}$; at c = 0.2, it ranges between $10^{-6}$ and $10^{-8}$; and at c = 0.4, it ranges between $10^{-7}$ and $10^{-8}$. The NN-BLM method coincides with the analytical answer, demonstrating the flawless modeling of a surrogate model. The figures show that the concentration of $H^{+}$ ions increases quickly from its initial value to its steady-state value. It is also clear that dimensionless $OH^{-}$ concentration steadily drops to a steady-state value of zero. Table 1 and Table 2 display the absolute differences between results provided by the NN-BLM algorithm for various instances and the desired data [46,47,48,49,50].

## 4. Results and Discussion

The figures of the numerical solutions of Equations (24) and (25) for dimensionless $H^{+}$ and $OH^{-}$ ion concentrations were constructed using Matlab software. The network was trained with the backpropagation Levenberg–Marquardt algorithm (BLMA). The NN-BLM technique is simple and has a straightforward framework for dealing with and processing nonlinear situations. The NN-BLMA is a gradient-free approach with a substantially faster convergence rate than other machine learning algorithms and cutting-edge approaches. It contains 70% (701 samples) training data, 15% (150 samples) validation data, and 15% (150 samples) testing data. Ten neurons were used in the fitting network’s hidden layer, as shown in Figure 3. Each neuron contained three weights, and the number of weights increased with the number of neurons. Table 3 displays the parameter settings for carrying out the design plan.

**Figure 7:**
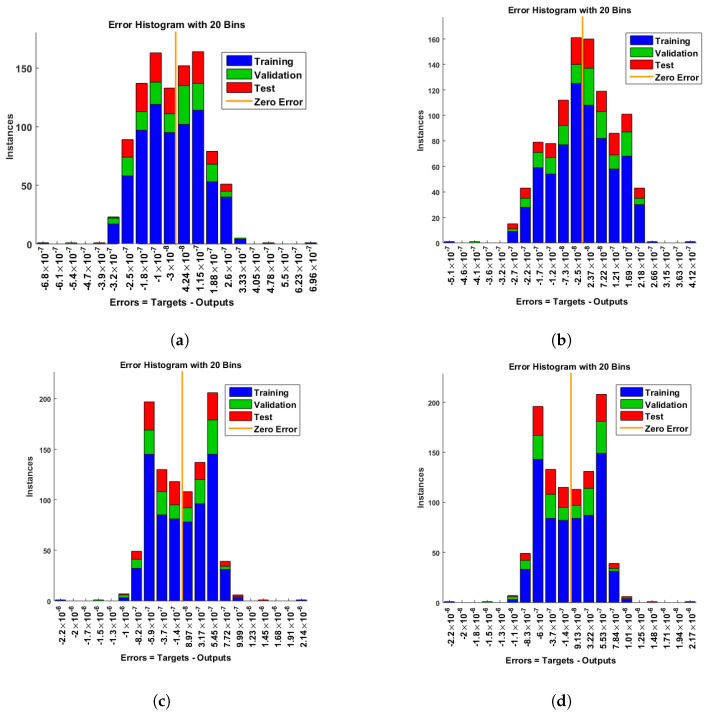
Error histogram analysis of dimensionless $H^{+}$ ion concentration. (**a**) At c = 0.1; (**b**) At c = 0.2; (**c**) At c = 0.3; (**d**) At c = 0.4.

**Figure 8:**
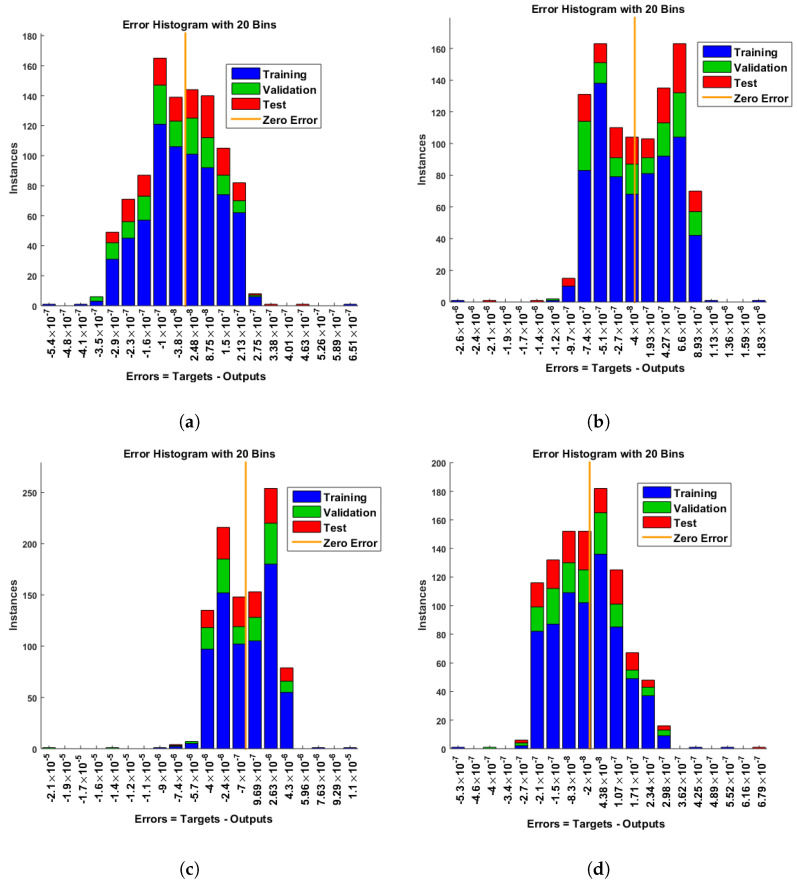
Error histogram analysis of dimensionless $OH^{-}$ ion concentration. (**a**) At c = 0.1; (**b**) At c = 0.2; (**c**) At c = 0.3; (**d**) At c = 0.4.

**Figure 9:**
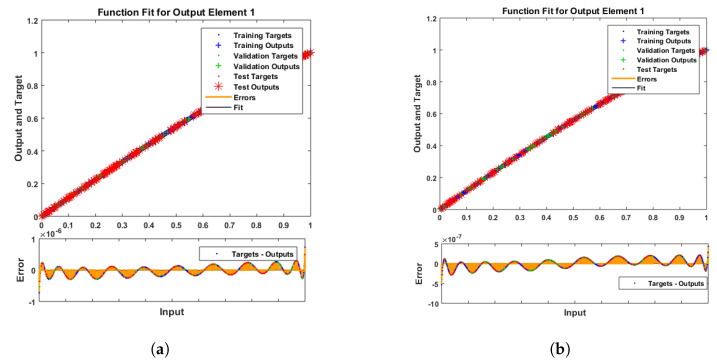

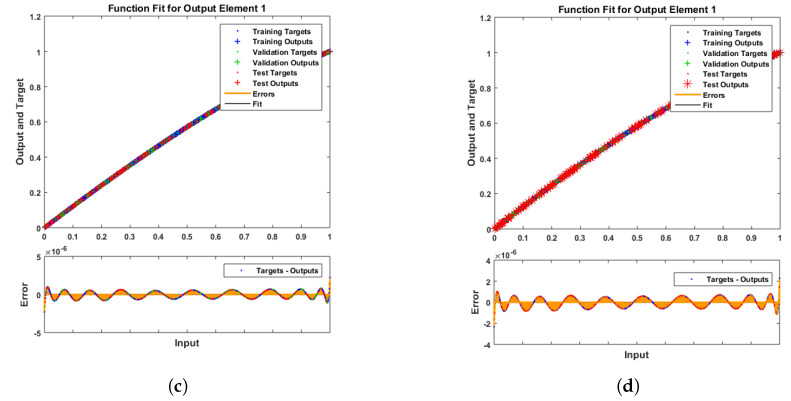
Fitting analysis of dimensionless $H^{+}$ ion concentration. (**a**) At c = 0.1; (**b**) At c = 0.2; (**c**) At c = 0.3; (**d**) At c = 0.4.

**Figure 10:**
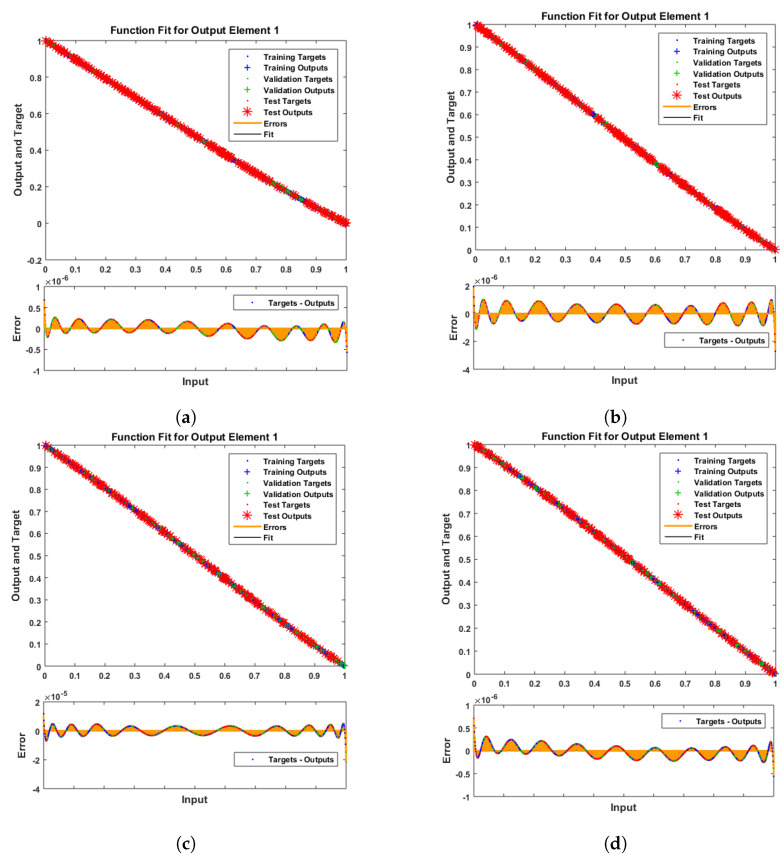
Fitting analysis of dimensionless $OH^{-}$ ion concentration. (**a**) At c = 0.1; (**b**) At c = 0.2; (**c**) At c = 0.3; (**d**) At c = 0.4.

**Figure 11:**
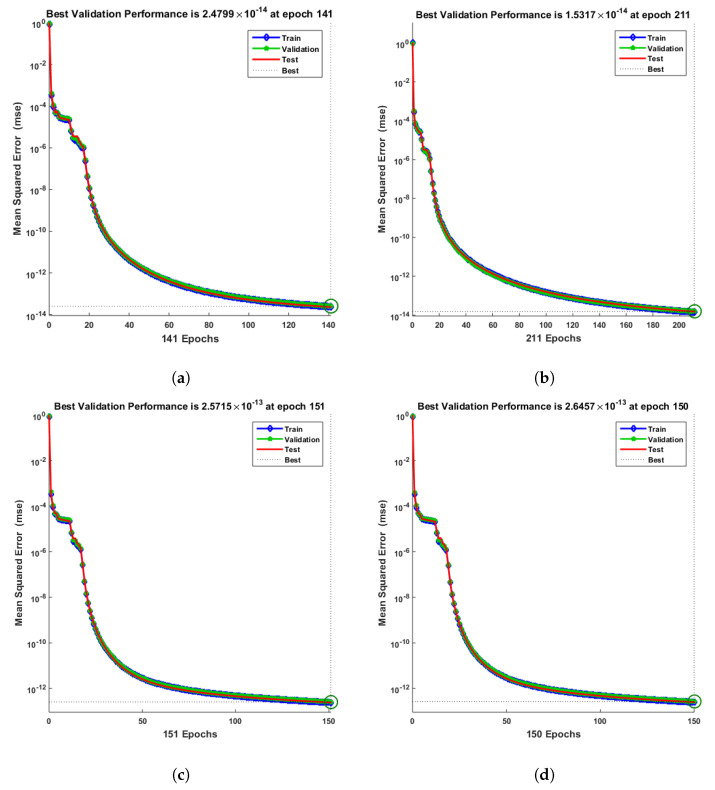
Mean-square error of NN-LMT prediction of dimensionless $H^{+}$ ion concentration. (**a**) At c = 0.1; (**b**) At c = 0.2; (**c**) At c = 0.3; (**d**) At c = 0.4.

**Figure 12:**
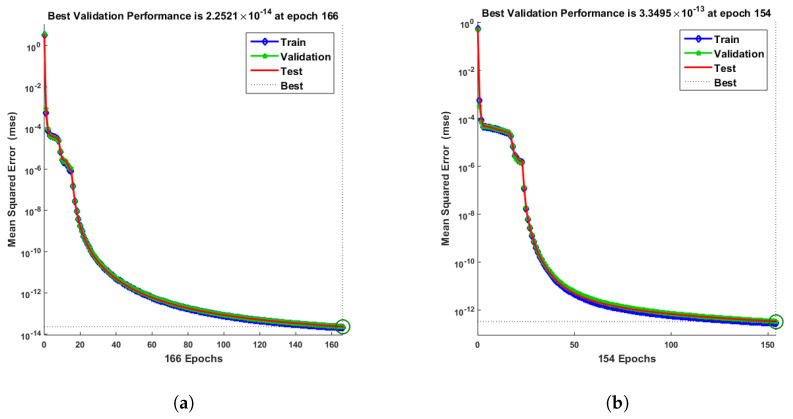

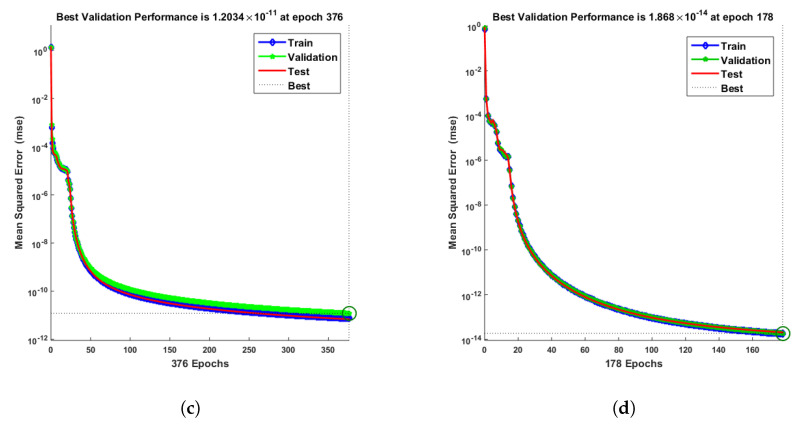
Meansquare error of NN-LMT prediction of dimensionless $OH^{-}$ ion concentration. (**a**) At c = 0.1; (**b**) At c = 0.2; (**c**) At c = 0.3; (**d**) At c = 0.4.

**Figure 13:**
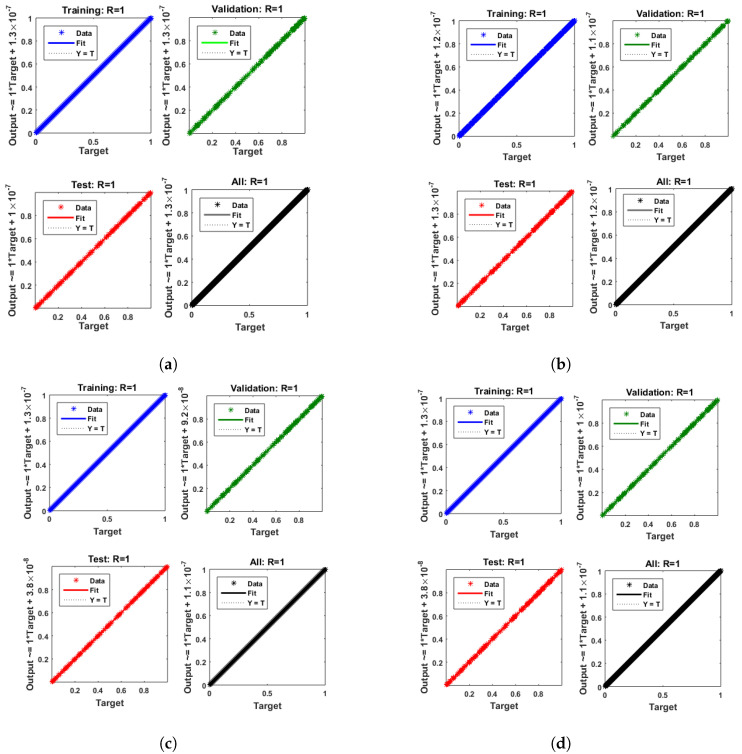
Regressionanalysis of dimensionless $H^{+}$ ion concentration. (**a**) At c = 0.1; (**b**) At c = 0.2; (**c**) At c = 0.3; (**d**) At c = 0.4.

**Figure 14:**
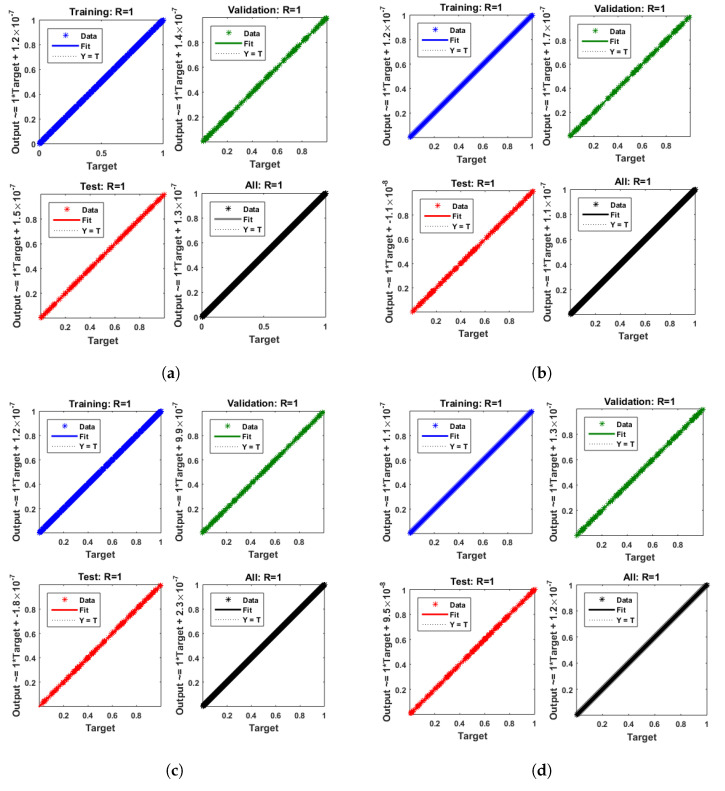
Regression analysis of dimensionless $OH^{-}$ ion concentration. (**a**) At c = 0.1; (**b**) At c = 0.2; (**c**) At c = 0.3; (**d**) At c = 0.4.

**Figure 15:**
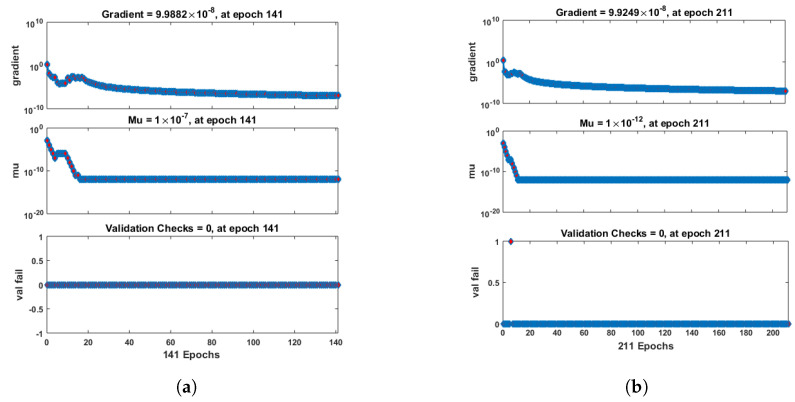

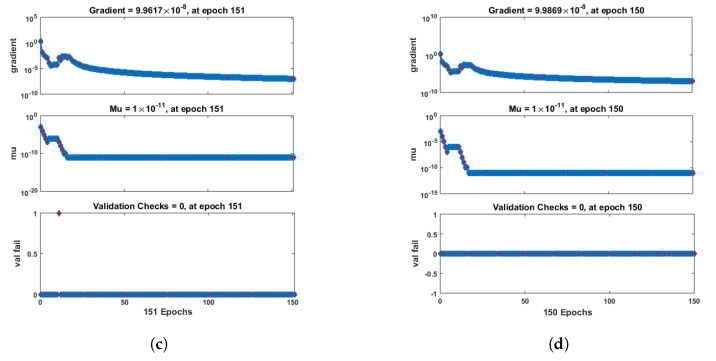
Performance analysis of dimensionless $H^{+}$ ion concentration. (**a**) At c = 0.1; (**b**) At c = 0.2; (**c**) At c = 0.3; (**d**) At c = 0.4.

**Figure 16:**
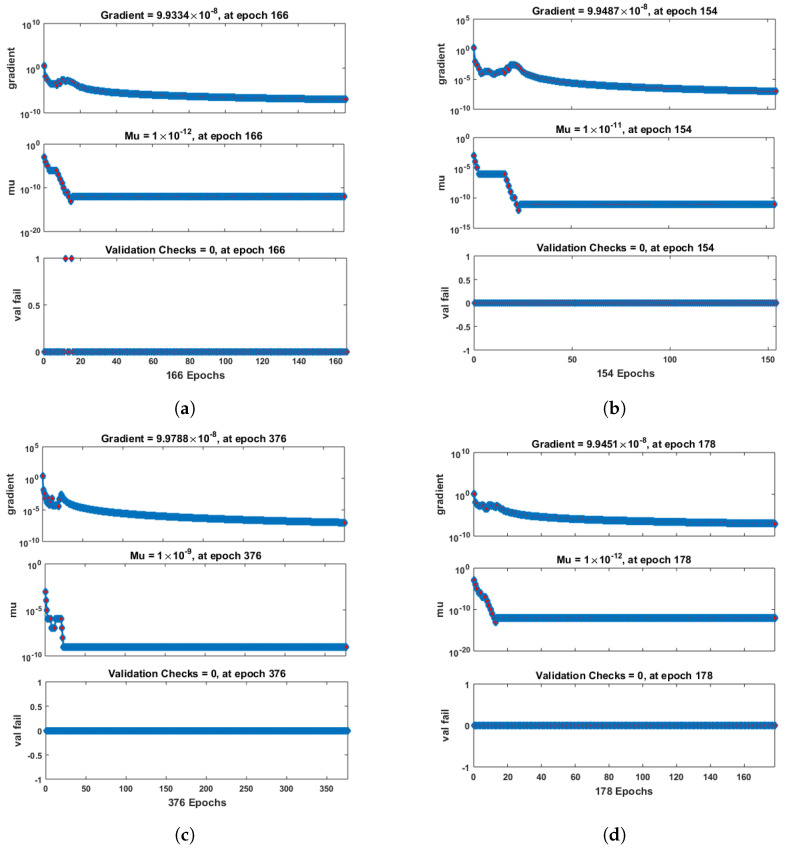
Performance analysis of dimensionless $OH^{-}$ ion concentration. (**a**) At c = 0.1; (**b**) At c = 0.2; (**c**) At c = 0.3; (**d**) At c = 0.4.

Figure 7 and Figure 8 shows that the approximate solution and targeted data of Equations (24) and (25) for dimensionless $H^{+}$ and $OH^{-}$ ion concentrations fit well together and have the fewest absolute errors. The absolute error (AE) for dimensionless $H^{+}$ concentration at c = 0.1 and c = 0.2 lies between $10^{-7}$ and $10^{-8}$; at c = 0.3 and c = 0.4, it lies between $10^{-6}$ and $10^{-8}$. The AE for dimensionless $OH^{-}$ ion concentration at c = 0.1 and c = 0.4 lies between $10^{-7}$ and $10^{-8}$; at c = 0.2, it lies between $10^{-6}$ and $10^{-8}$; and at c = 0.3, it lies between $10^{-5}$ and $10^{-7}$. Figure 9 and Figure 10 illustrate the fitting functions of Equations (24) and (25) for the non-dimensional $H^{+}$ and $OH^{-}$ ion concentrations at different values of the rate constant. Figure 9 shows that the non-dimensional $H^{+}$ ion concentration increases as the rate constant increases, and Figure 10 shows that the non-dimensional $OH^{-}$ ion concentration decreases progressively as the rate constant increases. Figure 11 and Figure 12 show the performance values of Equations (24) and (25) for the dimensionless $H^{+}$ and $OH^{-}$ ion concentrations. At c = 0.1, the best validation performance for the dimensionless $H^{+}$ ion concentration is $2.4799\times 10^{-14}$ at epoch 141; at c = 0.2, its value is $1.5317\times 10^{-14}$ at epoch 211; at c = 0.3, its value is $2.5715\times 10^{-13}$ at epoch 151; and at c = 0.4, its value is $2.6457\times 10^{-13}$ at epoch 151. At c = 0.1, for dimensionless $OH^{-}$ ion concentration, its value is $2.2551\times 10^{-14}$ at epoch 166; at c = 0.2, its value is $3.3495\times 10^{-13}$ at epoch 154; at c = 0.3, its value is $1.2034\times 10^{-13}$ at epoch 376; and at c = 0.4, its value is $1.868\times 10^{-13}$ at epoch 178. Figure 13 and Figure 14 show the regression analysis of Equations (24) and (25) for the non-dimensional $H^{+}$ and $OH^{-}$ ion concentrations for different values of the rate constant. Its value is one, which shows a close relationship between outputs and targets and the accuracy of the problem. Further, the statistical performance of the gradient of Equations (24) and (25) for the dimensionless $H^{+}$ and $OH^{-}$ ion concentrations at different values of a rate constant is illustrated in Figure 15 and Figure 16. At c = 0.1, for the dimensionless $H^{+}$ ion concentration, its gradient value is $9.9882\times 10^{-8}$ at epoch 141; at c = 0.2, its value is $9.9249\times 10^{-8}$ at epoch 211; at c = 0.3 its value is $9.9617\times 10^{-8}$ at epoch 151; and at c = 0.4, its value is $9.9869\times 10^{-8}$ at epoch 150. At c = 0.1, for the dimensionless $OH^{-}$ ion concentration, its gradient value is $9.9334\times 10^{-8}$ at epoch 166; at c = 0.2, its value is $9.9487\times 10^{-8}$ at epoch 154; at c = 0.3, its value is $9.9788\times 10^{-8}$ at epoch 376; and at c = 0.4, its value is $9.9451\times 10^{-8}$ at epoch 178. These figures also illustrate that mu in all cases lies between $10^{-7}$ and $10^{-12}$. Table 4 and Table 5 show the convergence metric for gradient, mu, epoch, testing, training, validation, and regression. From the above figures, it has been obtained that the dimensionless $H^{+}$ ion concentration rises quickly from a starting point to one at steady state, and the dimensionless $H^{+}$ ion concentration decreases progressively from a starting point to one at steady state. It is also implied that the $H^{+}$ ion concentration increases and $OH^{-}$ ion concentration decreases with increasing the rate constant.

## 5. Conclusions

In this study, the impacts of parameter variations in the mathematical model for diffusion of $OH^{-}$ and $H^{+}$ ions in the hydrogen production process in a non-buffered aqueous electrolyte were shown. Approximate solutions were calculated for the mathematical characterization of a rotating-disc electrode (RDE). This model contains a set of highly nonlinear, completely coupled equations. The nonlinear convection-diffusion equations were used, which were constructed using semi-boundary circumstances as an infinite notion. Reference solutions were found using the RK-4 numerical technique, and the outcome of NN-BLMA was contrasted with those of the reference solutions. The backpropagation Levenberg–Marquardt algorithm (BLMA) was used to train, test, and validate the calculated solution models. The profiles of the hydrogen ($H^{+}$) and hydroxide ($OH^{-}$) ion concentrations were calculated numerically. We displayed error histograms, absolute error, curve fitting graphs, and regression graphs of the dimensionless $H^{+}$ and $OH^{-}$ ion concentrations for different values of rate constant *c*. We also indicated how certain factors affect the amounts of hydrogen ($H^{+}$) and hydroxide ($OH^{-}$) ion concentrations at RDE. The numerically acquired data showed how the hydrogen evolution reaction system behaved. The $Rk_{4}$ and output results of the NN-BLMA were also compared with the help of Matlab software to see their behavior. The results show that the concentration of $H^{+}$ ions increases quickly from its initial value to its steady-state value, and that the dimensionless $OH^{-}$ ion concentration steadily drops to a steady-state value of zero. It is also implied that the $H^{+}$ ion concentration increases and $OH^{-}$ ion concentration decreases as the rate constant increases. This approach may be utilized for useful results for all hydrogen evolution models of rotating-disc electrodes (RDEs).

## Figures and Tables

**Table 1:** Dimensionless $H^{+}$ ion concentration.

	m(ζ)	${\mathrm{Rk}}_{4}$	BLMA	Error
At c = 0.1	0	0	$2.43\times 10^{-7}$	$2.43\times 10^{-7}$
	0.1	0.112628	0.112628	$7.65\times 10^{-8}$
	0.2	0.224126	0.224126	$7.99\times 10^{-8}$
	0.3	0.334163	0.334163	$3.91\times 10^{-8}$
	0.4	0.442201	0.442201	$2.97\times 10^{-10}$
	0.5	0.547518	0.547518	$5.56\times 10^{-9}$
	0.6	0.649234	0.649234	$6.17\times 10^{-9}$
	0.7	0.74636	0.74636	$6.25\times 10^{-8}$
	0.8	0.837858	0.837858	$1.15\times 10^{-7}$
	0.9	0.922711	0.922711	$1.09\times 10^{-7}$
	1	1	1	$2.20\times 10^{-7}$
At c = 0.2	0	0	$5.34\times 10^{-7}$	$5.34\times 10^{-7}$
	0.1	0.117043	0.117043	$1.60\times 10^{-7}$
	0.2	0.231951	0.231951	$1.57\times 10^{-7}$
	0.3	0.344387	0.344387	4.47$\times 10^{-8}$
	0.4	0.453808	0.453808	$9.72\times 10^{-8}$
	0.5	0.559495	0.559495	$2.60\times 10^{-8}$
	0.6	0.660585	0.660585	$6.33\times 10^{-8}$
	0.7	0.756128	0.756128	$3.61\times 10^{-8}$
	0.8	0.845145	0.845145	$1.55\times 10^{-7}$
	0.9	0.926705	0.926705	$1.92\times 10^{-7}$
	1	1	1	$4.36\times 10^{-7}$
At c = 0.3	0	0	$1.46\times 10^{-7}$	$1.46\times 10^{-7}$
	0.1	0.121457	0.121457	$1.16\times 10^{-7}$
	0.2	0.239776	0.239776	$1.04\times 10^{-7}$
	0.3	0.354611	0.354611	$7.20\times 10^{-8}$
	0.4	0.465415	0.465415	$2.60\times 10^{-8}$
	0.5	0.571471	0.571471	8.12$\times 10^{-10}$
	0.6	0.671937	0.671937	$2.42\times 10^{-8}$
	0.7	0.765896	0.765896	5.86$\times 10^{-8}$
	0.8	0.852433	0.852433	$8.15\times 10^{-8}$
	0.9	0.930699	0.930699	$8.29\times 10^{-8}$
	1	1	1	$1.40\times 10^{-7}$
At c = 0.4	0	0	$1.84\times 10^{-7}$	$1.84\times 10^{-7}$
	0.1	0.125872	0.125872	$1.10\times 10^{-7}$
	0.2	0.247601	0.247601	$1.04\times 10^{-7}$
	0.3	0.364835	0.364835	$6.84\times 10^{-8}$
	0.4	0.477022	0.477022	$2.68\times 10^{-8}$
	0.5	0.583448	0.583448	$4.50\times 10^{-9}$
	0.6	0.683288	0.683288	$2.75\times 10^{-8}$
	0.7	0.775665	0.775665	$5.40\times 10^{-8}$
	0.8	0.85972	0.85972	$6.86\times 10^{-8}$
	0.9	0.934694	0.934694	$6.24\times 10^{-8}$
	1	1	1	$1.25\times 10^{-7}$

**Table 2:** Dimensionless $OH^{-}$ ion concentration.

	n(ζ)	${\mathrm{Rk}}_{4}$	BLMA	Error
At c = 0.1	0	1	1	$1.62\times 10^{-7}$
	0.1	0.896201	0.896201	$1.10\times 10^{-7}$
	0.2	0.791525	0.791524	$9.64\times 10^{-8}$
	0.3	0.686286	0.686286	$5.69\times 10^{-8}$
	0.4	0.581012	0.581012	$2.07\times 10^{-8}$
	0.5	0.476435	0.476435	$4.28\times 10^{-10}$
	0.6	0.373469	0.373469	$1.36\times 10^{-8}$
	0.7	0.273176	0.273176	$4.27\times 10^{-8}$
	0.8	0.176716	0.176716	$7.91\times 10^{-8}$
	0.9	0.085278	0.085278	$8.91\times 10^{-8}$
	1	$-3.6\times 10^{-9}$	$1.3\times 10^{-7}$	$1.31\times 10^{-7}$
At c = 0.2	0	1	0.999998	$1.94\times 10^{-6}$
	0.1	0.900616	0.900615	$7.85\times 10^{-7}$
	0.2	0.79935	0.799349	$6.61\times 10^{-7}$
	0.3	0.69651	0.69651	$2.59\times 10^{-7}$
	0.4	0.592619	0.592619	$6.06\times 10^{-7}$
	0.5	0.488411	0.488411	$1.82\times 10^{-7}$
	0.6	0.38482	0.384819	$6.23\times 10^{-7}$
	0.7	0.282944	0.282944	$1.93\times 10^{-7}$
	0.8	0.184004	0.184004	$3.84\times 10^{-8}$
	0.9	0.089272	0.089272	3.53$\times 10^{-8}$
	1	$1.99\times 10^{-9}$	$2.7\times 10^{-6}$	$2.72\times 10^{-6}$
At c = 0.3	0	1	1	$2.53\times 10^{-7}$
	0.1	0.905031	0.90503	$1.13\times 10^{-7}$
	0.2	0.807175	0.807175	$1.04\times 10^{-7}$
	0.3	0.706734	0.706734	$2.45\times 10^{-8}$
	0.4	0.604225	0.604225	$2.15\times 10^{-8}$
	0.5	0.500388	0.500388	$1.05\times 10^{-8}$
	0.6	0.396172	0.396172	$5.29\times 10^{-10}$
	0.7	0.292713	0.292713	$4.35\times 10^{-8}$
	0.8	0.191291	0.191291	$8.11\times 10^{-8}$
	0.9	0.093267	0.093267	$7.17\times 10^{-8}$
	1	$4.9\times 10^{-9}$	$2.3\times 10^{-7}$	$2.24\times 10^{-7}$
At c = 0.4	0	1	0.999999	$7.11\times 10^{-7}$
	0.1	0.909445	0.909445	7.94$\times 10^{-8}$
	0.2	0.815	0.815	$8.51\times 10^{-8}$
	0.3	0.716958	0.716958	$6.50\times 10^{-8}$
	0.4	0.615832	0.615832	$1.58\times 10^{-7}$
	0.5	0.512365	0.512365	1.35$\times 10^{-8}$
	0.6	0.407523	0.407523	$6.56\times 10^{-8}$
	0.7	0.302481	0.302481	$5.64\times 10^{-8}$
	0.8	0.198578	0.198578	$1.48\times 10^{-7}$
	0.9	0.097261	0.097261	$1.70\times 10^{-7}$
	1	$8.01\times 10^{-9}$	$5.7\times 10^{-7}$	$5.60\times 10^{-7}$

**Table 3:** Parameters for the NN-BLM algorithm’s implementation.

Training	Testing	Validation	Max.iteration	Hidden Neurons	Performance Function
70%	15%	15%	1000	10	Mean square error

**Table 4:** NN-BLMA’s performance measurement statistics for different rate constant values to obtain dimensionless $H^{+}$ ion concentration solutions.

Mean Square Error								
**c**	**Neurons**	**Epochs**	**Gradient**	**Mu**	**Training**	**Testing**	**Validation**	**Regression**
0.1	10	141	$9.99\times 10^{-8}$	1.00$\times 10^{-07}$	$2.40\times 10^{-14}$	$2.31\times 10^{-14}$	$2.48\times 10^{-14}$	1
0.2	10	211	$9.93\times 10^{-8}$	$1.00\times 10^{-12}$	$1.25\times 10^{-14}$	$1.52\times 10^{-14}$	$1.53\times 10^{-14}$	1
0.3	10	151	$9.96\times 10^{-8}$	$1.00\times 10^{-11}$	$2.42\times 10^{-13}$	$2.42\times 10^{-13}$	$2.57\times 10^{-13}$	1
0.4	10	150	$9.99\times 10^{-8}$	$1.00\times 10^{-11}$	$2.50\times 10^{-13}$	$2.51\times 10^{-13}$	$2.65\times 10^{-13}$	1

**Table 5:** NN-BLMA’s performance measurement statistics for different rate constant values to obtain dimensionless $OH^{-}$ ion concentration solutions.

Mean Square Error								
**c**	**Neurons**	**Epochs**	**Gradient**	**Mu**	**Training**	**Testing**	**Validation**	**Regression**
0.1	10	166	$9.93\times 10^{-8}$	$1.00\times 10^{-12}$	$2.09\times 10^{-14}$	$2.32\times 10^{-14}$	$2.25\times 10^{-14}$	1
0.2	10	154	$9.95\times 10^{-8}$	$1.00\times 10^{-11}$	$2.94\times 10^{-13}$	$3.40\times 10^{-13}$	$3.35\times 10^{-13}$	1
0.3	10	376	$9.98\times 10^{-8}$	$1.00\times 10^{-09}$	$7.54\times 10^{-12}$	$6.82\times 10^{-12}$	$1.20\times 10^{-11}$	1
0.4	10	178	$9.95\times 10^{-8}$	$1.00\times 10^{-12}$	$1.79\times 10^{-14}$	$2.06\times 10^{-14}$	$1.87\times 10^{-14}$	1

## Data Availability

Not applicable.

## Nomenclature

**Symbol**	**Description**	**Unit**
$C_{H^{+}}$	$H^{+}$ ion concentration	mol cm^−3^
$C_{OH^{-}}$	$OH^{-}$ ion concentration	mol cm^−3^
$C_{H^{+}}^{\infty }$	$H^{+}$ ion bulk concentration	mol cm^−3^
$C_{OH^{-}}^{\infty }$	$OH^{-}$ ion bulk concentration	mol cm^−3^
$D_{H^{+}}$	coefficient of diffusion of $H^{+}$ ions	cm^−2^ s^−1^
$D_{OH^{-}}$	coefficient of diffusion of $OH^{-}$ ions	cm^−2^ s^−1^
υ	kinematic viscosity	cm^2^ s^−1^
Ω	rotation rate	s^−1^
$k_{+3}$	forward rate coefficient	mol^−1^ s^−1^ cm^3^
$k_{-3}$	backward rate coefficient	mol s^−1^ cm^−3^
$v_{z}$ = −0.51 $z^{2}$ $\Omega^{\frac{3}{2}}$ $v^{\frac{-1}{2}}$	velocity	cm s^−1^
a = 0.51023 $v^{\frac{-1}{2}}$ $\Omega^{\frac{3}{2}}$	parameter	cm^−1^ s^−1^
$m_{0}$ = $\frac{k_{-3}}{D^{\frac{1}{3}}a^{\frac{2}{3}}C_{H^{+}}^{\infty }}$	dimensionless backward rate coefficient	none
$m_{1}$ = $\frac{k_{+3}C_{H^{+}}^{\infty }}{D^{\frac{1}{3}}a^{\frac{2}{3}}}$	dimensionless forward rate coefficient	none
m = $-m_{1}$ uv + $m_{0}$	parameter	none
ψ	current	Ampere (or) C s^−1^
η	potential	volt
F	Faraday constant	C mol^−1^
A	area	cm^−2^
T	temperature	K
ζ	dimensionless axial distance	none
Z	axial distance	cm
